# Geographical Distribution of the Space-Weaving Spider, *Chibchea salta*, from Northwestern Argentina: New Records and Bioclimatic Modeling

**DOI:** 10.1673/031.011.5401

**Published:** 2011-04-21

**Authors:** Gonzalo D. Rubio, Luis E. Acosta

**Affiliations:** CONiCET, Cátedra de Diversidad Animal I, Facultad de Ciencias Exactas, Físicas y Naturales, Universidad Nacional de Córdoba, Av. Vélez Sarsfield 299, X5000JJC Córdoba, Argentina

**Keywords:** Bioclim, bioclimatic profile, ecological niche modeling, Maxent, presence-only, Yungas Ecoregion

## Abstract

New records of the spider *Chibchea salta* Huber 2000 (Araneae, Pholcidae) from northwestern Argentina are provided, and the potential range of this species is modeled. Two presence-only methods, Maxent and Bioclim, were run using 19 bioclimatic parameters at a resolution of 30 arc seconds. The climatic profile of *C. salta* is described, and the relative importance of the bioclimatic variables is explored. Temperature variables proved to be more decisive to the final range shape. The range predicted with Maxent is slightly larger than with Bioclim, but the latter appears to be more sensitive to the record set bias. Both methods performed well, resulting in predictive ranges consistent with the yungas ecoregion. These results provide an initial insight into the bioclimatic tolerance of *C. salta*, and by identifying potential areas with no records, such as the sierras on the Salta-Jujuy border, they also help in identifying sites for future sampling efforts.

## Introduction

Research on spiders in Argentina has been hitherto mainly restricted to taxonomic and systematic work on several families, together with a few papers referring to community and diversity studies (Liljesthröm et al. 2002; Beltramo et al. 2006; Avalos et al. 2007; Rubio et al. 2008), but none concerning the study of species responses to climate and its variation in space. Much work is still needed to understand the distribution patterns of the native fauna of spiders; and this would be especially meaningful in areas with an added conservation value (e.g. the yungas ecoregion), due to the biological diversity and the gradual loss of many of their habitats. At present, studies on Araneae aimed to tackle biogeographical questions are completely lacking in Argentina. Research at local and regional scales suggest, however, that spiders are well suited for biogeographic studies, since they are strongly influenced by the habitat type and other environmental parameters, so that their presence is fairly predictable (Uetz 1975; Weeks and Holtzer 2000; Pinkus-Rendón et al. 2006).

Novel methods that estimate species potential distributions by combining observed occurrences with environmental variables proved their effectiveness for application across a range of biogeographical analyses (Maes et al. 2005; Pearson et al. 2007). These kinds of analyses use the facilities provided by the Geographical Information Systems (GIS), and allows obtaining a first empiric approach to the range of ecophysiologic tolerance through the evaluation of the bioclimatic profile, by identifying the environmental conditions and areas in which a given species would be able to survive (Pearson 2007). Such predictive models have been proposed as useful tools to supplement incomplete data of distribution of species (Maddock and Du Plessis 1999; Raxworthy et al. 2003; Acosta 2008). All these methods assume that the resulting predictive distribution model is a function of the way in that species respond to the environmental variables, thus reflecting a subset of their fundamental niche (Austin 2002). Predictive distribution patterns and their inherent bioclimatic profiles provide valuable information for numerous ecological-environmental applications; for example, to detect areas where intensive sampling would be worthwhile or necessary, to define conservation priorities, or to predict potential biological invasions (Guisan and Zimmermann 2000; Raxworthy et al. 2003; Graham and Hijmans 2006; Ward 2007; Acosta 2008; Giovanelli et al. 2008). An extensive array of modeling algorithms is available to investigate relationships between predictor variables and species presence-only datasets (Guisan and Zimmermann 2000; Guisan and Thuiller 2005). Two of these methods, Bioclim (Fischer et al. 2001; Walther et al. 2004), and Maxent (Phillips et al. 2006) are among the most popular algorithms in the literature and have proven good performance and accuracy for these kinds of studies (Elith et al. 2006; Hijmans and Graham 2006; Ward 2007; Echarri et al. 2009; Rubio et al. 2010); both are well suited to the aims of this study.

The purpose of this paper is to provide new records and to model the potential distribution of the spider, *Chibchea salta* Huber 2000 (Pholcidae), that is characteristic of a high diversity area in northwestern Argentina, the “yungas” ecoregion. It is also aimed to visually compare the resulting prediction maps obtained with the two above-mentioned modeling methods. One major objective of this study was to explore the climatic profile underpinning the species distribution, in order to start gathering preliminary knowledge on its niche requirements — a fact almost completely ignored for the vast majority of Neotropical spiders. *Chibchea* is a relatively small pholcid genus, currently comprised of 16 nominal species (Platnick 2009). This genus spreads over a wide range on the west of South America, from Colombia and Venezuela up to northern Argentina and Chile. It is apparently restricted to the Andean corridor, where it can inhabit over 3500 masl (e.g. *C. abiseo* from Perú) (Huber 2000). All species of *Chibchea* are small to medium-sized spiders, generally with dark colors and globose to oval, higher-than-long opisthosoma. This is the only *Chibchea* species known in Argentina, and has been recorded in just two localities within the humid subtropical yungas forests in Salta Province. It belongs to a clade that represents the southernmost distribution of the whole genus, also containing species from Peru and Bolivia: *C. aberrans, C. araona, C. uru, C. silvae* and *C. malkini* (Huber 2000).

**Table 1.**
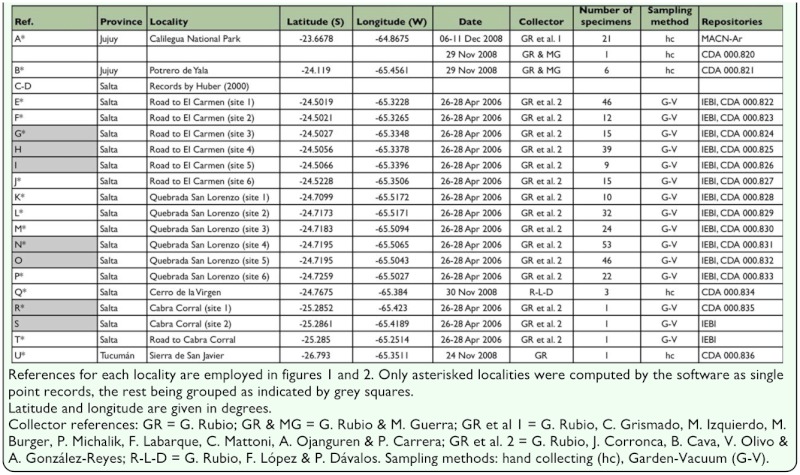
New records of *Chibchea salta*, with geographical coordinates and sampling details

**Figure 1.**
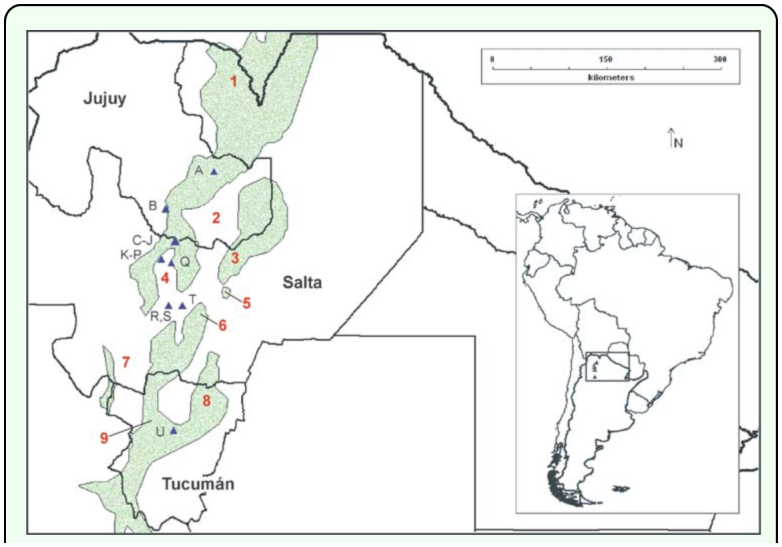
Locality records of *Chibchea salta* Huber 2000 (blue triangles), and extent of the yungas montane forest ecoregion (green area) in northwestern Argentina (from Olson et al. 2001). References: 1: Baritú National Park; 2: San Francisco Valley; 3: El Rey National Park; 4: Lerma Valley in Central Salta; 5: Sierra de la Lumbrera; 6: Sierras de Metán; 7: Valles Calchaquies; 8: Sierra de Medina; 9: Sierra del Aconquija/Cumbres Calchaquies. Localities (letters) are as listed in Table 1. Inset: location of the map area in South America. High quality figures are available online.

Modeling the distribution of a yungas spider has an additional importance. The referred ecoregion has long been recognized to have high endemicity and biodiversity rates, in Argentina only matching the Paranaense forests in Misiones Province (Brown et al. 2002). Elevation determines three definite vegetation belts in the yungas, well defined by the plant species composition and physiognomy: (1) pedemontane rainforests, (2) montane rainforests and (3) montane forests (Brown et al. 2002, 2006). The yungas ecoregion extends as a narrow strip for more than 4000 km on the eastern slopes of Andean and sub-Andean mountains (Cabrera and Willink 1973). The Argentinean portion represents the southernmost end. In this country this ecoregion is discontinuous and split into patches (Acosta 2002; Brown et al. 2006). In the Salta Province, at approximately 25° 10′ S, the Chaco ecoregion (a xeric thornforest) ingresses into the Valle de Lerma, causing the main disruption of the yungas and its fragmentation into patches (Figure 1), thus creating a complex interface with marked contrasts in short distances. According to Brown et al. (2006) this central sector is an important connectivity area with high-priority for conservation. *Chibchea salta* inhabits this central sector in Salta, hence the importance of this study that utilizes a novel approach for Argentinean spiders.

## Methods

### Species occurrence records

All available records of *C. salta* were used, including the few references in the literature (just two records from Salta Province: 17 km N of La Caldera, -24.5030 S -65.3351 W, and 22 km N of La Caldera, -24.5011 S -65.3180 W: Huber 2000) together with our own records (Table 1). Most of the latter were obtained in an ongoing ecological study in different sites in the central portion of Salta Province using the Garden-Vacuum method (Bolger et al. 2000; Bell et al. 2002) to collect spiders on vegetation. Additional samples were obtained from other yungas sectors: Jujuy and Salta Provinces for the northern yungas, and Tucumán representing the southern part. Records were georeferenced either *in situ*, using a Map-60 Garmin-GPS, or with the use of different digital gazetteers available in the Internet (mainly Google Earth ©). This dataset was arranged to be used within a geographic information system (DIVA-GIS 5.4, Hijmans et al. 2005a). The complete dataset consisted of 21 point records (Table 1), but due to duplicate records from the same gridcell being removed by the software during the analysis, valid effective records were restricted to 15 points. The specimens examined were deposited in the following Argentinean institutions (abbreviations and curators in parentheses): Instituto para el Estudio de la Biodiversidad de Invertebrados, Universidad Nacional de Salta (IEBI, J.A. Corronca); Colección Nacional Aracnológica, Museo Argentino de Ciencias Naturales “Bernardino Rivadavia” (C. Scioscia and M. Ramírez); and Colección Aracnológica de la Cátedra de Diversidad Animal I, Facultad de Ciencias Exactas, Físicas y Naturales, Universidad Nacional de Córdoba (L.E. Acosta).

**Table 2.**
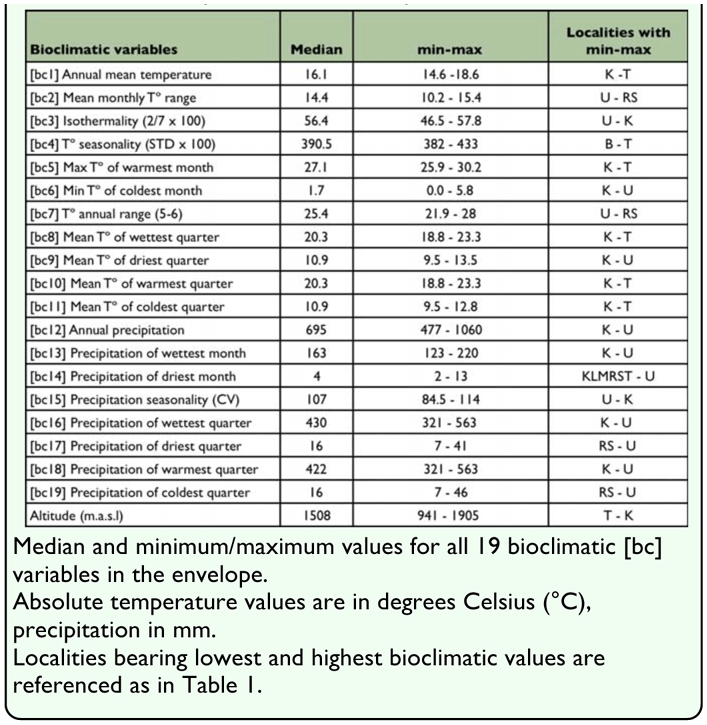
Summary of the bioclimatic profile of Chibchea salta:

### Environmental data

Values of 19 bioclimatic parameters were extracted from the WorldClim database (Hijmans et al. 2005b) at a resolution of 30 arc-seconds (∼1 km^2^). These parameters (bioclimatic variables) fall into two broad categories — temperature or precipitation variables — and are listed in Table 2. Elevation data were available for reference purposes, but not used as a predictor by themselves.

### Modeling methods

The geographical range of *C. salta* was modeled with two widely used, presence-only methods, Maxent and Bioclim. The Maxent algorithm (described in detail by Phillips et al. 2006) yields results ranging from 0 to 1, indicating relative suitability of a given grid cell (high values indicate a higher presence probability) (Graham and Hijmans 2006; Phillips et al. 2006). Since probability is continuous, a threshold needs to be set to separate suitable from not suitable gridcells; in this study, the “maximum training sensitivity plus specificity” threshold rule was applied (Liu et al. 2005). Other relevant settings of the software were used in their default values, including the convergence threshold (10^-5^); maximum background points (10,000); maximum iterations (1,500); replicated run type (subsample), output format (logistic), and “auto features” activated. Resulting predictions were visualized and mapped by importing the ASCII files into DIVA-GIS 5.4 grid format (Hijmans et al. 2005a). Version 3.3.0 of the Maxent software was employed (Phillips et al. 2009). To estimate the relative contribution of each variable in the final model a jackknife analysis was applied as a built-in functionality of Maxent (Phillips et al. 2006). The Bioclim model was built using its implementation in DIVA-GIS 5.4 (Hijmans and Graham 2006; Ward 2007; Acosta 2008; Echarri et al. 2009). Bioclim is a frequency distribution based algorithm, which extracts values of each bioclimatic variable from all localities and arranges them in a cumulative frequency distribution. The set of values of all variables defines the bioclimatic profile of the species, delimiting the so called “envelope”, i.e. the climatic conditions that bound all occurrence localities (Guisan and Zimmermann 2000; Fischer et al. 2001; Walther et al. 2004; Ward 2007). In the potential distribution maps, gridcells are scored as suitable (if within the envelope; i.e. the presence of the species can be expected) or unsuitable (if outside the envelope) (Acosta 2008). The “most limiting factor” analysis, available in Bioclim, was applied to detect, for a given gridcell, which variable is most critical to the inclusion of that gridcell within the resulting envelope.

### Model evaluation

For Bioclim, the original data set was split and a subset of 30% presence points was set apart as a test sample. Pseudo-absence points were generated from the background using DIVA-GIS. The model was then run using the remaining 70% of the original presence data (training sample), randomly resampled in 20 repetitions (Maes et al. 2005; Pearson 2007; Acosta 2008; Echarri et al. 2009). Models obtained in these repetitions where overlaid to get a first visual evaluation of their mutual consistence and with the model built using the full dataset. Subsequently, the accuracy of the model was evaluated by calculating the AUC (area under curve) in a receiver operating characteristic plot, and the maximum Kappa (max-*k*). AUC values vary from 0.5 (model not better than random) to 1.0 (perfect accuracy as indicative that the model can discriminate perfectly between presences and absences of records); the max-*k* values over 0.75 are deemed to be excellent (Louto et al. 2005; Graham and Hijmans 2006; Randin et al. 2006). In the case of Maxent models, the program routinely calculates the AUC for each run.

### Additional localities sampled

To empirically test whether localities in which the species was proven to be absent are correctly classed by the models as unsuitable, additional samplings were carried out in sites that are close to the record localities but correspond to a different ecoregion, the Chaco thorn-forest. These extra samples were obtained in central Salta using the above mentioned Garden-Vacuum method (i.e. with identical sampling effort). They consisted of 11 sites (Figures 9–10): General Güemes (three sampling sites at -24.6562 S -65.0035 W; -24.654 S -64.9917 W; -24.653 S -64.9892 W), along Juramento River (three sites: 25.0894 S -65.0025 W; -25.1345 S -65.0093 W; -25.1676 S -64.9861 W), near Cabra Corral dam (three sites: -25.1219 S -65.0361 W; -25.1205 S -65.0574 W; -25.1209 S 65.0921 W), south of La Merced (-25.0501 S 65.4962 W), and Castellanos (-24.7194 S 65.4367 W). The Chaco ecoregion is characterized by xeric and semi-deciduous forests, with shrub and herbaceous strata as well; all localities except one (General Güemes) belong to the “Sierra chaco” sub-ecoregion, extended on the basal slopes of the mountains. Sierra chaco interdigitates among yungas patches, having thus an important role in the connectivity of the northern and southern yungas sectors (Brown et al. 2006). As stated, in all 11 Chaco localities *C. salta* was not recorded (Figures 9–10).

## Results

### New records of *Chibchea salta*

Nineteen new localities for this species, along with collection information and geographical coordinates are listed in Table 1.

### Bioclimatic profile

**Figure 2.**
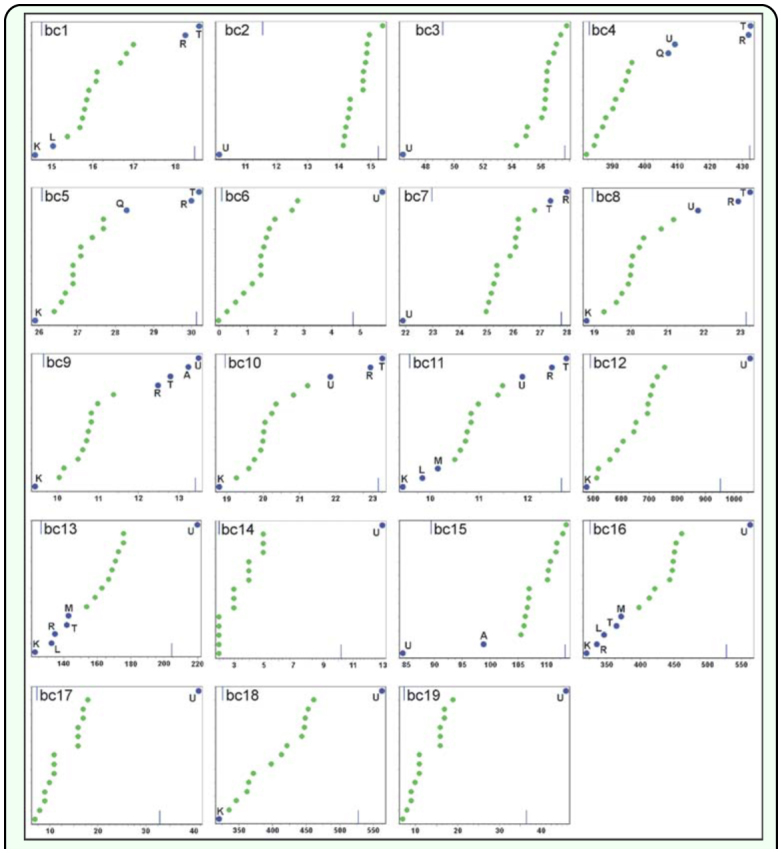
Bioclimatic profile of *Chibchea salta:* for each bioclimatic variable, cumulative relative frequencies (0–100) are displayed for the full data set. Blue dots indicate outlier localities, their references are the same as listed in Table 1. High quality figures are available online.

The envelope of *C. salta*, as obtained with Bioclim, contains (with the default percentile threshold of 0.025) 66.7% of the presence records (i.e. 10 of 15 points fall within all possible bidimensional variable combinations). Table 2 summarizes the bioclimatic profile of this species by indicating minimum, maximum, and medians for all 19 bioclimatic variables. Quebrada de San Lorenzo, site 1 (K in Table 1 and Figure 1) has the highest score of extreme bioclimatic values of the envelope (n = 14). Many of these extreme values indicate this locality as the coldest and driest site (bel, 5–6, 8–14, 16, 18), as well as with highest isothermality and precipitation seasonality (bc3, 15); all these features were consistent with the elevation of the locality, the highest in the whole dataset (1905 m). Another locality with many extreme bioclimatic values (13 variables) is site U (San Javier, Tucumán), in this case showing the highest precipitation (bc12–14, 16–19), and lowest values of isothermality and temperature range (bc2–3, 7; see also Figure 2). Site T (road to Cabra Corral, in Salta Province), representing the record with lowest elevation and closest to the Chaco plains east of the mountains, is the warmest place (bel, 5, 8, 10–11) and has highest temperature seasonality (bc4; Figure 2), principally due to the high values in December and January.

### Potential range

**Figure 3–4.**
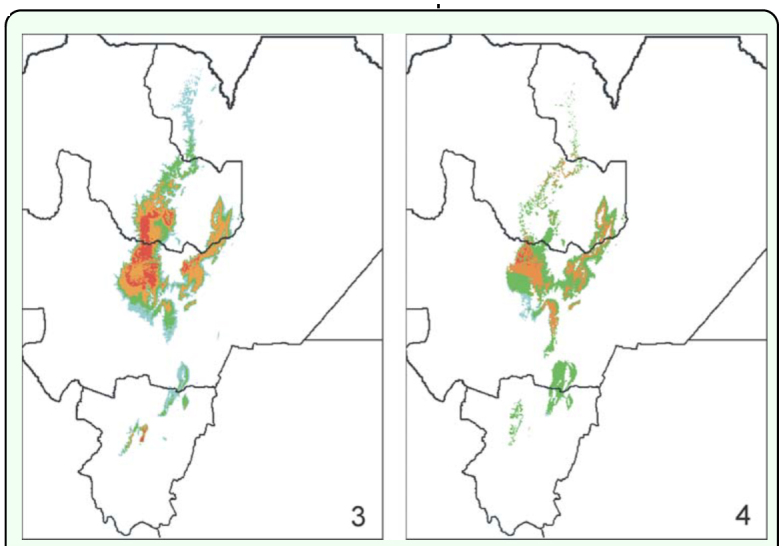
Predicted range of *Chibchea salta*, as resulted in the Maxent (Figure 3) and Bioclim (Figure 4) analyses using all 19 bioclimatic variables and the full dataset. Code colors indicate either climatic or habitat suitability: Maxent (Figure 3, shown as probability): red (0.90–1), orange (0.80–0.90), green (0.65–0.80), light blue (0.50–0.65); Bioclim (Figure 4, as cumulative distribution percentile): red (20–34), orange (10–20), green (5– 10), light blue (2.5–5). High quality figures are available online.

Models obtained with Maxent and Bioclim overall share a similar pattern (Figures 3–4), and both, in turn, match fairly well the yungas ecoregion (Figure 1). The highest probability (Maxent) or suitability (Bioclim) is consistently situated around the central area of Salta, i.e. where most records originate (Figure 1). The area with highest climatic suitability (0.80–1) recognized by Maxent is larger, extending from central Salta (Lerma Valley, San Lorenzo) to mid-southern Jujuy (Figure 3). In contrast, the highest suitability in the Bioclim model is much more concentrated in the Lerma Valley up to San Lorenzo, but reaches southern Jujuy only weakly (Figure 4). In both models the predicted range extends southwards, bordering northern and eastern slopes near Metán sierras. Bioclim and Maxent also agree in detecting one relevant potential area on the East, with no records of the species yet, close to the El Rey National Park and along the mountain group on the Jujuy-Salta border (Figures 1, 3–4). This area comprises several contiguous Sierras, interestingly bearing a large easternmost yungas isolate; it includes a small isolated mountain as well (Sierra de la Lumbrera), also recovered in isolation in the models (Figures 1, 3–4). Although with different intensity, both models project the species range north- and southwards. The projection into northern Salta (near the Baritú National Park area) is more continuous though with low probability in Maxent (Figure 3), whereas it is just represented by scattered dots in Bioclim (Figure 4). The southwards extension into Tucuman Province is separated from the core area in both models, and predicts one larger zone on the Northeast (Sierra de Medina) and a narrow strip on east faced slopes of the Aconquija-Calchaquíes range (Figures 3–4); only the Maxent model gives high probability on the surroundings of the sole record in that province (site U, Sierra de San Javier; Figure 1), while Bioclim remarkably ranks the latter as a marginal site bearing, as stated above, many extreme values for bioclimatic variables.

### Limiting factors and relative importance of variables

**Figure 5–6.**
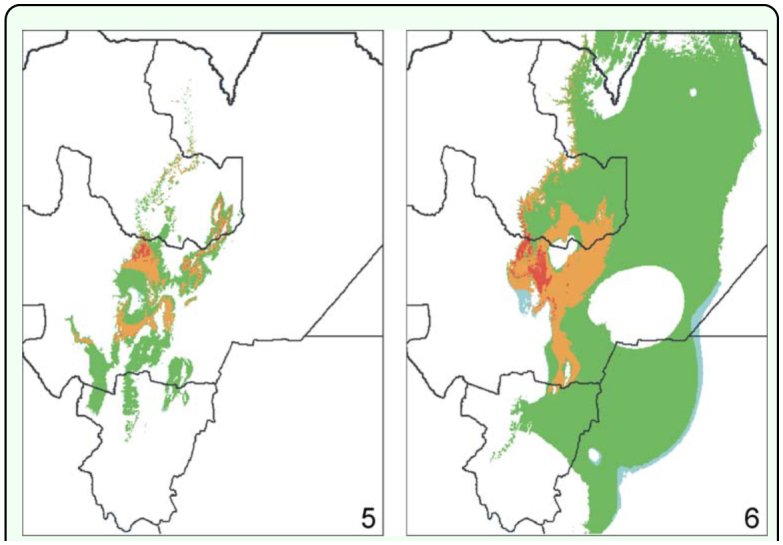
Potential distribution of *Chibchea salta* modeled with Bioclim, only with temperature variables (bc 1-bc 11; Figure 5) or precipitation variables (be 12-be 19; Figure 6), using the full dataset. Color codes are the same as those listed in Figures 3 and 4. High quality figures are available online.

Separate models were built in Bioclim with either temperature (be 1–11) or precipitation variables (be 12–19), to investigate their relative contribution to the final model (Figures 5–6). Results show that precipitation variables are clearly restrictive on the West because of the decrease of rainfall (following the increase of elevation), and partly on the South — mainly in the sub-xeric Lerma Valley in Salta Province. On the contrary, these variables are remarkably permissive towards the East into the Chaco ecoregion (part of Santiago del Estero, eastern Salta, even entering the Bolivian territory: Figure 5), showing that precipitation in this area would be enough for the species. Models obtained with temperature variables alone (be1–11) result in a much narrower area more similar to the final model, but still with a remarkable permisiveness into xeric mountain valleys (mainly the “Valles Calchaquíes” region, between Salta and Tucumán Provinces; Figures 1, 5) where no montane forest exists. Temperature variables are critical to the species range mostly on the southeastern and northern part of the predicted range (presumably related to a latitudinal gradient), and towards the East, in this case preventing geographical expansion into the warm (though otherwise humid enough) Chaco plains (Figures 5–6). For example, bc6 (minimum temperature of the coldest month) is the most limiting factor on the margins of the San Francisco Valley with Chaco vegetation (Jujuy; Figure 1), and a small move into the valley represents an increase of >3° C for this variable which makes it to fall outside the species envelope; bc6 is also restrictive in southern Salta and northern Tucumán where the increase can be of 5° C when moving apart from the core area into the Chaco. From another viewpoint, the jackknife analysis performed in the Maxent run indicated that three variables linked to temperature (bc5: maximum T° of warmest month, bc6: minimum T° of coldest month, and be 10: mean T° of warmest quarter) and one of precipitation (bc15: precipitation seasonality) are the most relevant when the range is considered as a whole, i.e. they show the highest gain when analyzed individually; bc6 is the variable that decreases the gain the most when omitted (Figure 8). These results emphasize the major importance of temperature variables in the final models, constraining the climatic niche within a quite narrow thermic tolerance (cf. Table 2).

### Model performance and comparisons

Both Maxent and Bioclim performed well and their resulting modeled ranges are consistent to each other (Figures 3–4). The range predicted with Maxent is 35% larger than with Bioclim, showing that in the latter, suitable gridcells tend to concentrate more around record points. The overlay of the resulting maps of 20 runs in Bioclim using training data (70% of stochastic original records) are highly consistent with the predicted range using all points. Bioclim values of AUC were of high accuracy (0.80–0.97; mean = 0.93, 20 replicates using training data), while max-*k*; proved excellent performance in average (0.61–0.99; mean = 0.86, same number of replicates). In Maxent, AUC values resulting from the training data were excellent (0.998– 0.999; mean = 0.998, 20 repetitions). These AUC values should be taken with caution, however, since they might be over-rated due to the low number of records. Aside from the statistical meaning, the modeling proved to be in reasonable agreement with the expected range, especially considering the distribution of yungas formations and the type of environment inhabited by this species (mountain forests and rainforests, G.D.R. pers. obs.). Although this study did not focus primarily on absence data, samplings available for 11 Chaco sites yielded no specimen of *C. salta* outside the predicted range (Figures 9–10). All Chaco sites placed east of the mountains (i.e. in the Chaco plains proper; C–D on Figures 9–10) matched negative areas of the models that reflects a correct prediction in this sector. This is especially remarkable for the row of sites along Juramento River (D) that seems to follow a narrow unsuitable corridor between suitable gridcells. These observations strongly suggest the models ability to make correct predictions on negative areas. However, this ability did not stand the same for Chaco sites placed on the west, inside the Lerma Valley: with Maxent both sites (Castellanos and south of La Merced: A–B on Figure 9) fall not only within the predicted positive area, but with high probability; in the Bioclim model, the former site was classed among presence gridcells too, though the latter (actually placed on the very limits of the suitable area) did not.

## Discussion

As in other cases (Elith et al. 2006; Pearson et al. 2007; Ortiz-Martínez et al. 2008; Boubli and de Lima 2009;), the bioclimatic analysis proved to be a valuable means to get insight of the fundamental niche features of a species with still scarce records and almost no previous ecological knowledge. Both methods performed consistently well considering that a narrow-ranged yungas species — probably a true endemics species — was used. As shown elsewhere (Rubio et al. 2010), not every yungas-dwelling spider is necessarily endemic to this ecoregion, but very little is known so a general pattern cannot be drawn. It is interesting to note that, although models were built with selected climatic variables alone, the resulting prediction redraws quite well the yungas ecoregion that is defined by vegetation physiognomy and composition. Models did not explicitly include a vegetation constraint, but vegetation is generally assumed to be a critical determinant in the presence or absence of most pholcids. In that sense, the correct prediction of absence, as matched in most Chaco sites where *Chibchea salta* was “absent”, gives an additional support of the soundness of the models. This predictive value is in general appreciated as a remarkable strength of ecological niche modeling (Muñoz et al. 2009).

In accordance with results obtained for a harvestman species (Acosta 2008), temperature has proven to be more determinant to the final range shape—of *Chibchea salta*, assuming sufficient humidity. In this case, the modeled area has meaningful altirudinal differences in short distances, so that both temperature and precipitation gradients consist of step changes that limit distribution on east and west sides. This close relationship of the range of *C. salta* and elevation (actually by means of the climatic gradient determined thereby) is common to other yungas taxa as well, as evidenced by the already mentioned altitudinal belts of this ecoregion (Brown et al. 2006). These features are no doubt well depicted in the models due to the fine grain resolution used (Acosta 2008).

Potential distributions yielded by two quite different methods, Bioclim and Maxent, are fairly consistent. As commonly suggested, no single modeling method is thought to have the complete truth (Elith et al. 2006; Stockman et al. 2006; Ward 2007), so that comparative analyses like this may help to gain a better understanding. Ranges obtained with Bioclim, despite its lower computational performances, probably look biogeographically more realistic for a species that has been observed to be closely dependent on humid forests. This is best exemplified in the Lerma Valley in Salta Province, where Maxent gives high probability to some areas known to be covered by Chaco vegetation; this portion, in contrast, was only weakly predicted in Bioclim, in better accordance to known biological facts. In turn, all Chaco localities east of the sierras (General Güemes, Juramento River) were correctly assigned as negative in both models. The correct assignment of unsuitability along the Juramento corridor is remarkable, since these Chaco sites are placed in a geographically intricate region where striking contrasts can be observed over very small distances (there are several sectors where opposite slopes at a single point may differ sharply, bearing yungas vegetation on one side and Chaco on the other). It seems clear that the climatic conditions in the Lerma Valley — surrounded by mountains instead of being freely exposed to eastern air masses and humidity — are peculiar, and might eventually compromise the accuracy of the climatic layers (generated by extrapolation; Hijmans et al. 2005b), leading the models to wrong predictions in that area.

In any case, Bioclim appears to be more sensitive to the record set bias and to concentrate more in areas where point density is higher. It proved to be especially sensitive to outliers, as shown in the northern and southern portions of the potential range, hardly recovered by this method despite the fact that actual records are available (see Acosta 2008 for similar results). Both Bioclim and Maxent agree in detecting a presumable high suitability area, where the species has not been recorded yet: the isolated sierras group on the East, near the El Rey National Park. This is an extensive though still little surveyed yungas sector, and our results clearly point to it as a priority area to be targeted in future sampling efforts. These results are thus not deemed to be a complete picture of the range and the climatic niche of this spider, but rather provide a starting point for further research.

**Figure 7–8.**
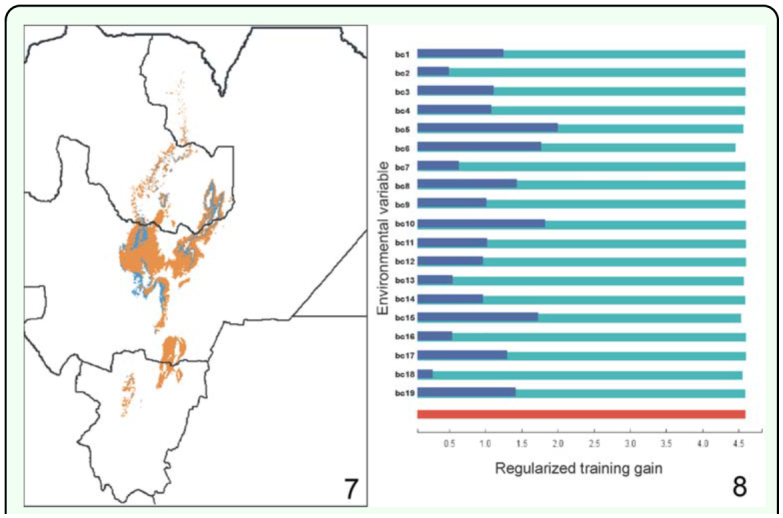
Contribution of bioclimatic variables to the final model. Figure 7: Most limiting factors analysis of *Chibchea salta* using Bioclim; gridcells where temperature variables are limiting are colored in orange, those limited by precipitation variables are in blue. Figure 8: Jackknife of the regularized training gain (Maxent model): without variable (light blue), with only variable (blue), with all variables (red). High quality figures are available online.

**Figure 9–10.**
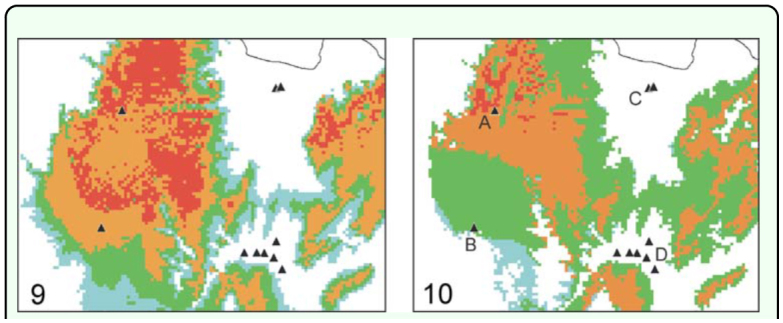
Close-up of the central portion of the predicted range of *C. salta* (between parallels -24.51° and -25.28° S) showing the position of all 11 Chaco sites (black triangles) available for assessment of the negative predictions. They are contrasted with the areas predicted by Maxent (Figure 9) and Bioclim (Figure 10). References: A = Castellanos, B = near La Merced (A and B placed in the Lerma Valley), C = General Güemes, D = Juramento River and road to Cabra Corral Dam. Remark: in Figure 10, point B actually falls inside a negative gridcell, though the scale used does not allow this fact to be easily seen. High quality figures are available

## Abbreviations

**AUC**,: area under curve;
**bc**,: bioclimatic variable;
**GIS**,: Geographical Information Systems
